# Levels of Cadmium in Human Mandibular Bone

**DOI:** 10.3390/toxics7020031

**Published:** 2019-06-04

**Authors:** Andrew W. Browar, Landon L. Leavitt, Walter C. Prozialeck, Joshua R. Edwards

**Affiliations:** 1 College of Dental Medicine – Illinois, Midwestern University, 555 31st Street, Science Hall, Room 211-J, Downers Grove, IL 60515, USA; lleavitt83@midwestern.edu; 2 Department of Pharmacology, College of Graduate Studies, Midwestern University, Downers Grove, IL 60515, USA; wprozi@midwestern.edu (W.C.P.); jedwar@midwestern.edu (J.R.E.)

**Keywords:** cadmium, bone, mandible, periodontal disease, bioaccumulation, body burden, gender differences, One Health

## Abstract

Cadmium (Cd) is an environmental toxicant that accumulates in bone and alters bone turnover and metabolism. Periodontal disease is characterized by tooth loss and tissue destruction, specifically, loss of supporting bone around the teeth. We have previously shown that Cd causes loss of dental alveolar (tooth supporting) bone in a rodent model of long-term Cd poisoning. The overall goal of this study was to determine the possible association between levels of Cd in alveolar bone and evidence of periodontal disease in human cadavers. The extent of Cd accumulation in human mandible samples was analyzed. Levels of Cd in mandibular alveolar bone were compared to those in basal bone as well as the renal cortex in samples obtained from the cadavers. Alveolar bone contained significantly higher levels of Cd when compared to basal bone (*p* < 0.01). Cd levels in mandibular bone were significantly higher in female compared to male cadavers (*p* < 0.05). The kidney cortex had greater than 15-fold higher Cd levels compared to mandible bone. Additional analyses showed a possible association between levels of Cd in basal bone and the presence of periodontal disease in cadavers from which the samples were obtained. This study shows that Cd accumulates to relatively high levels within alveolar bone as compared to basal bone in the mandible and thus may have a significant and direct effect in the progression of changes in bone associated with periodontal disease.

## 1. Introduction

Cadmium (Cd) is a well-known environmental pollutant that causes damage to a variety of organs, including kidneys, testes, liver, lung, and bone. Cd exposure has long been known to be associated with the development of osteomalacia and osteoporosis (for a review, see [1]). Exposure to Cd can occur from foods, environmental and industrial exposure, and with smoking, including second hand smoke [2,3,4,5]. Cd may have indirect adverse effects on bone by causing renal dysfunction that results in increased levels of urinary calcium and vitamin D [6]. There is also evidence that Cd causes direct osteotoxicity in humans [7] and in animal models of Cd exposure (for a review, see [8]).

Alveolar (tooth supporting) bone loss is a result of the progression of periodontal disease that eventually leads to tooth loss [9]. There is some evidence that Cd exposure is associated with periodontal disease. However, there are conflicting epidemiological studies that show or fail to show a connection between Cd and periodontal disease (for a review, see [10]). We have recently published a report showing that Cd causes loss of alveolar bone in a rodent model of long-term Cd poisoning [10].

The purpose of this study was to determine the levels of Cd within mandibular alveolar and basal bone as well as the kidney cortex from human cadavers and to examine a possible correlation between Cd content in mandibular bone and periodontal disease scores.

## 2. Materials and Methods

### 2.1. Sample Collection

De-identified human cadavers donated to Midwestern University were used in this study, and so the study did not require formal institutional review. A total of 12 cadavers were selected, 6 in the first round of sample collection and 6 in a second round. The samples selected for this study were only from cadavers that had teeth in the anterior of the mandible. As such, a new group of cadavers was needed following the first round of selection so that one year separated rounds one and two for cadaver selection. Periodontal crevice depths were measured using a Williams periodontal probe (Hu-Friedy). After the assessment of probing depths, each cadaver was assigned a periodontal score between 1 and 4: (1) No sign of periodontal involvement, (2) probing depth 2–4 mm, (3) probing depth 4–6 mm, (4) probing depth >6 mm [11].

**Figure 1:**
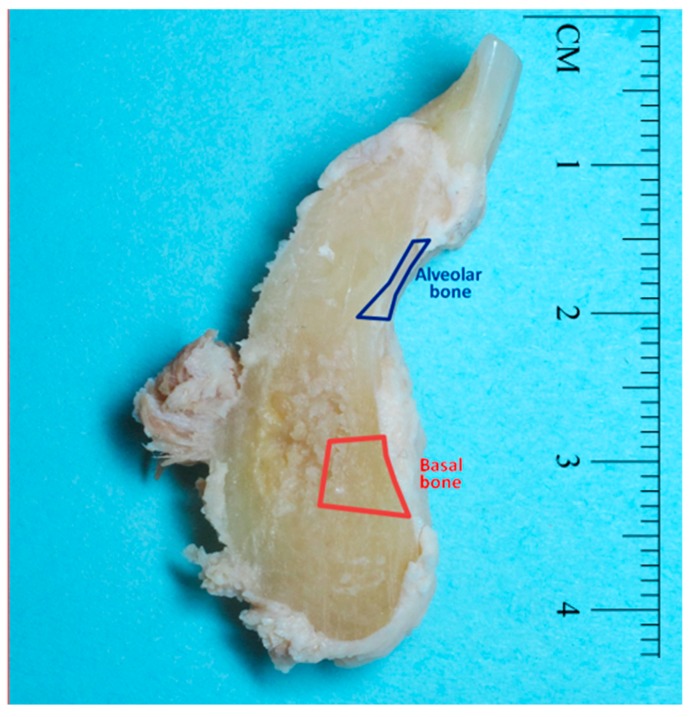
Image showing a representative human mandible jaw section after gross dissection where alveolar bone (blue outline) and basal bone (red outline) samples were harvested to be analyzed for cadmium (Cd) content.

Mandibular bone samples were harvested to include 2 to 3 teeth from the anterior mandible and including the full height of the mandible (Figure 1). Kidney cortex samples were also harvested from these cadavers and sent for analysis of Cd content.

Mandibles were processed in our laboratory. Soft tissue was removed from the teeth and bone with periodontal instruments. Samples of alveolar bone (blue outline in Figure 1) and basal bone (red outline in Figure 1) were further dissected to be tested separately for Cd content.

**Figure 2:**
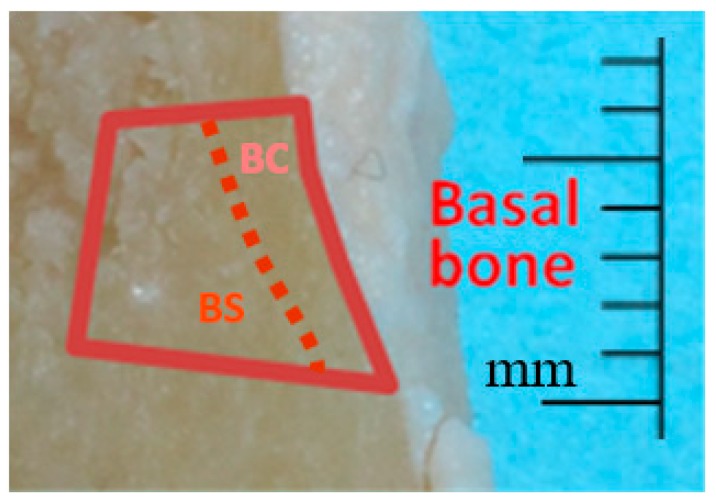
Image showing a representative human mandible jaw section after gross dissection where basal cortical bone samples (BC) and basal spongy bone samples (BS) were harvested to be analyzed for Cd content.

In a subsequent round of analysis of mandibular bone, 6 of the 12 mandible samples were selected that had Cd levels in the middle of the range from the previous basal bone analysis. These were further dissected to separately harvest basal cortical bone and spongy (trabecular) bone (Figure 2). These samples were then analyzed for Cd content.

### 2.2. Cd analysis

Kidney cortex and bone samples were digested in nitric acid and analyzed for Cd by inductively coupled plasma mass spectrometry at Chemical Solutions LTD (Harrisburg, PA, USA). The limit of detection was 0.01 μg-Cd/g-bone dry weight. For samples with a reported value of ≤0.01 μg-Cd/g-(below limit of detection) bone dry weight, the number was divided by 2 and 0.005 was used in calculating mean values and performing statistical analyses.

### 2.3. Statistics

Potential significant differences in Cd content between alveolar and basal bone were determined using a paired *t*-test while correlations between Cd content of various tissues or periodontal scores were performed using Pearson’s correlation analysis with the Graph Pad Prism (v. 7.04) statistical program (La Jolla, CA, USA). For all analyses, *p* ≤ 0.05 was considered statistically significant. All data are expressed as mean ± standard error.

## 3. Results

A total of 12 cadaver subjects were included in the study. The average age was 69.8 ± 4.6 years overall with the mean age for males (*n* = 8) being 72.3 ± 5.4 years and females (*n* = 4) 65.5 ± 8.8 years. There was no medical history or tobacco use data available for the cadavers that were used in this study; only gender and age were known.

**Figure 3:**
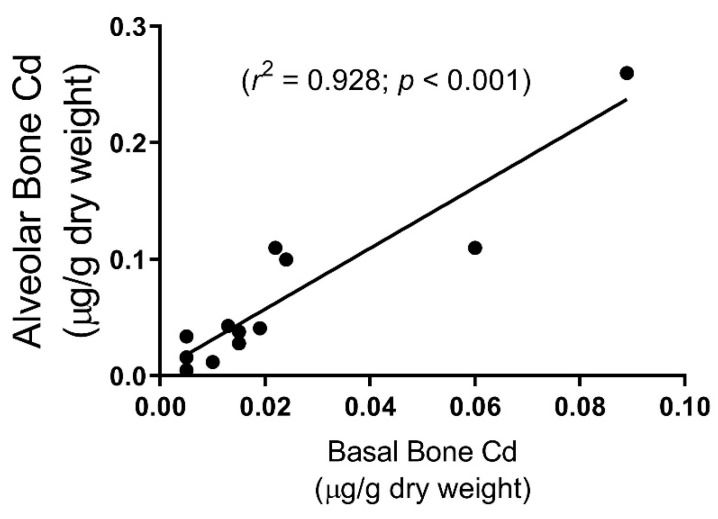
A significant and positive correlation exists in Cd content between pairs of alveolar and basal mandible bone samples in human cadavers (*n* = 12). Four values were below the limit of detection (<0.01 µg/g Cd dry weight)—one subject in both alveolar and basal bone, and two in basal bone only.

Mandibular alveolar bone samples showed significantly higher levels of Cd (0.06 ± 0.02 μg-Cd/g-bone dry weight) as compared to basal bone samples (0.02 ± 0.01, *p* = 0.01) (Table 1). There was a large range in Cd accumulation between samples (from <0.01 (limit of detection) to 0.26), however, when comparing alveolar bone to basal bone Cd levels within each pair of samples from the same cadaver, there was a very high correlation (*p* < 0.001) (Figure 3). Four values were below the limit of detection (<0.01 µg/g Cd dry weight)—one subject in both alveolar and basal bone, and two in basal bone only.

**Figure 4:**
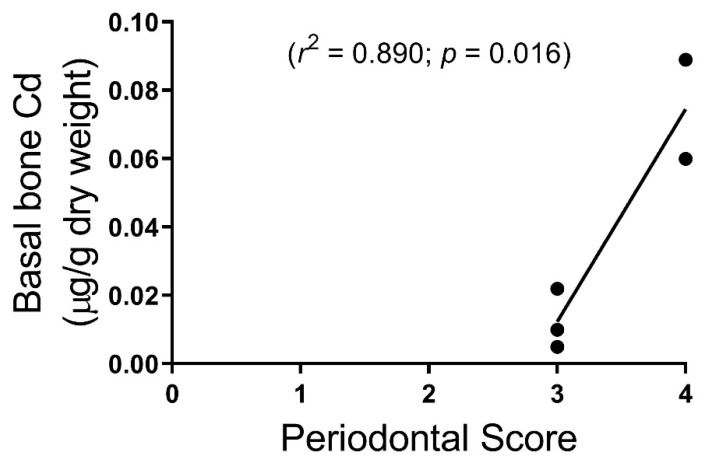
Human cadaver periodontal scores were correlated with basal mandible bone Cd content (*n* = 5).

Periodontal scores showed a correlation with alveolar bone Cd content but not to statistical significance (*p* = 0.16). Periodontal scores correlated with basal mandible bone Cd content (*p* = 0.016) (Figure 4). Only five subjects were scored from the first round of six cadavers. We could not reach a consensus on the sixth subject, which scored between 3 and 4. Therefore, only five were included and then none from the second round of six cadavers. It is important to note that the periodontal scores from the cadavers were inconsistent because of limited access due to rigor mortis, fixation, and desiccation of cadavers; so, while a statistically significant correlation was found between Cd levels in basal bone and periodontal scores, further studies on live subjects are needed to determine if this correlation is valid.

In comparing cortical bone to spongy bone samples for Cd content, there was no statistical difference between them (Table 1). There was no correlation between age and Cd levels in bone or kidney cortex.

There was no apparent difference in kidney cortex Cd levels between male (1.97 ± 0.50; *n* = 4) and female subjects (1.55 ± 0.03; *n* = 2). Only the first round of cadavers (*n* = 6) were analyzed for Cd in the kidney cortex, since no apparent relationship was found between kidney cortex Cd levels and any other measurable value (Table 1). In agreement with other reports [12], we found that the Cd content of the kidney cortex was greater than 15-fold higher than that of bone samples.

## 4. Discussion

While the literature shows several studies relating the prevalence of periodontal disease to exposure to environmental Cd (for a review, see [10]), this is the first study to examine the bioaccumulation of Cd in the human mandible. Cd bone levels found in human mandible samples were in a similar range to Cd levels found in transilliac bone biopsies on patients with end-stage renal disease [13]. Tissue Cd content reported here is per gram dry weight while several other authors have reported Cd per gram tissue wet weight. The following equation is used to determine µg/g gram tissue wet weight values: ((100 – % tissue water)/100) multiplied by µg/g tissue dry weight. The kidney has a higher water content at approximately 75% compared to bone at 22%. Thus, the range of Cd in the kidney cortex is 0.056 to 2.4 µg/g wet weight; this is a fairly low value considering the age of the individuals. This would imply that individuals experience Cd exposure that is higher than that reported in the current study, or those with occupational exposure would likely have an even higher risk of developing periodontal disease [13]. While there was a great range in Cd accumulation between samples in this study, it is notable that within each pair of samples, Cd levels in alveolar bone were significantly greater than its paired basal bone sample (Figure 3).

Effects of Cd exposure on bone metabolism have been shown in several studies (for a review, see [10]). These Cd effects can be direct as with changes in blood calcium regulatory hormones [7], or osteotoxic effects on osteoblasts [8]. There is also evidence of indirect effects of Cd on bone. Cadmium exposure in experimental animals has been shown to induce an immune response similar to that found in diabetes, cardiovascular disease, and periodontal disease [14,15,16,17,18,19,20]. In addition, renal dysfunction following Cd exposure may have secondary effects on bone health. In one epidemiologic study, Cd exposure at a level not resulting in proximal tubule dysfunction did not change forearm bone mineral density in post-menopausal women [21]; suggesting that Cd-induced renal dysfunction is required for bone mineral density loss. On the other hand, the renal levels of Cd in the present study are relatively low, well below the threshold level that is associated with the onset of kidney injury [12]. This issue is confounded by the fact that no data was available regarding the history of kidney disease and Cd exposure in the subjects. Further studies are needed to resolve these issues.

Initially, in this study, we set out to correlate the periodontal status of the cadaver subjects to Cd accumulation. We did see a trend that showed higher Cd content in mandible samples correlated with poorer periodontal scores (Figure 4), however, we found that data collection for periodontal scores in this model were incomplete, inconsistent, and did not reflect live patients due to rigor mortis and desiccation of gingival tissue. Although not ideal, the use of embalmed and non-embalmed human cadavers to investigate periodontal disease and oral health has been reported [22,23].

Our results show significant gender differences in Cd levels in alveolar as well as basal mandibular bone. This finding is remarkable considering the low ‘*n*’ values of the current data set. Others have reported gender-specific differences on the potentially direct osteotoxic effects of Cd [7] as well as mediators of bone metabolism [24]. In this study, it should be noted that all female cadavers were assumed to be post-menopausal with an age range of 52 to 91 years. One explanation for this gender difference is that women are more likely than men to be iron-deficient and this condition may result in a greater Cd body burden over a lifetime [25,26]. However, no obvious gender difference was observed in Cd levels in the kidney cortex in this study. This would suggest that while the body burden of Cd in the renal cortex is by far the greatest, Cd levels in the mandible bone are more reflective of factors, such as iron status and other gender differences.

Considering that each of the paired samples showed greater Cd levels in alveolar bone than basal bone, this would lead one to understand that Cd exposure affects alveolar bone differently than basal bone, even in subjects with minimal Cd levels. Previous studies examining bone turnover in the mandible have shown that alveolar bone is very dynamic because of the function of tooth support and the periodontal ligament [27,28]. A study comparing proteoglycans content of alveolar bone vs. basal bone in experimental rabbits showed significantly higher levels in alveolar bone, suggesting a higher turnover of alveolar bone compared to basal bone, and alveolar bone being more metabolically active [29]. Our finding of a greater accumulation of Cd in alveolar bone suggests that Cd may have more direct osteotoxic effects in this bone as compared to other types of bone.

Further research is needed in the clinical setting to verify the results shown here that Cd exposure is associated with periodontal disease and that these Cd effects on periodontal disease are due to direct osteotoxic effects on the alveolar jawbone.

## Figures and Tables

**Table 1:** Cadmium content of human cadaver samples subdivided by bone type and gender.

	Cadmium µg/g Dry Weight	
Alveolar Bone *n* = 12	^#^ 0.06 ± 0.02	
	Male *n* = 8	0.03 ± 0.01
	Female *n* = 4	* 0.13 ± 0.05
Basal Bone *n* = 12	0.02 ± 0.01	
	Male *n* = 8	0.01 ± 0.01
	Female *n* = 4	* 0.04 ± 0.02
	Cortical *n* = 6	0.04 ± 0.01
	Spongy *n* = 6	0.03 ± 0.01
Kidney Cortex *n* = 6	1.83 ± 0.45	
	Male *n* = 4	1.97 ± 0.50
	Female *n* = 2	1.55 ± 0.03

A number sign (#) indicates significant differences between alveolar bone compared to basal bone (paired, two-tailed t-test; *p* < 0.01). An asterisk (*) indicates significant differences (unpaired, two-tailed *t*-test; *p* ≤ 0.05) between male and female. Collective basal and alveolar bone samples had an ‘*n*’ value of 12; male and female samples were 8 and 4, respectively. Kidney cortex had a total *n* value of 6 with only 2 samples from females so no statistical analysis was performed. Data are mean ± SE.

## References

[1] Nordberg G.F. (2009). Historical perspectives on cadmium toxicology. Toxicol. Appl. Pharmacol..

[2] Satarug S., Swaddiwudhipong W., Ruangyuttikarn W., Nishijo M., Ruiz P. (2013). Modeling cadmium exposures in low- and high-exposure areas in Thailand. Environ. Health Perspect..

[3] Jarup L., Akesson A. (2009). Current status of cadmium as an environmental health problem. Toxicol. Appl. Pharmacol..

[4] Paschal D.C., Burt V., Caudill S.P., Gunter E.W., Pirkle J.L., Sampson E.J., Miller D.T., Jackson R.J. (2000). Exposure of the U.S. population aged 6 years and older to cadmium: 1988–1994. Arch. Environ. Contam. Toxicol..

[5] Böhlandt A., Schierl R., Diemer J., Koch C., Bolte G., Kiranoglu M., Fromme H., Nowak D. (2012). High concentrations of cadmium, cerium and lanthanum in indoor air due to environmental tobacco smoke. Sci. Total Environ..

[6] Uchida M., Teranishi H., Aoshima K., Katoh T., Kasuya M., Inadera H. (2007). Elevated urinary levels of vitamin D-binding protein in the inhabitants of a cadmium polluted area, Jinzu River basin, Japan. Tohoku J. Exp. Med..

[7] Schutte R., Nawrot T.S., Richart T., Thijs L., Vanderschueren D., Kuznetsova T., Van Hecke E., Roels H.A., Staessen J.A. (2008). Bone resorption and environmental exposure to cadmium in women: A population study. Environ. Health Perspect..

[8] Bhattacharyya M.H. (2009). Cadmium osteotoxicity in experimental animals: Mechanisms and relationship to human exposures. Toxicol. Appl. Pharmacol..

[9] Oliver R.C., Brown L.J. (1993). Periodontal diseases and tooth loss. Periodontol 2000.

[10] Browar A.W., Koufos E.B., Wei Y., Leavitt L.L., Prozialeck W.C., Edwards J.R. (2018). Cadmium Exposure Disrupts Periodontal Bone in Experimental Animals: Implications for Periodontal Disease in Humans. Toxics.

[11] Armitage G.C. (2003). Position Paper: Diagnosis of Periodontal Diseases. J. Periodontol..

[12] Satarug S., Baker J.R., Reilly P.E., Moore M.R., Williams D.J. (2002). Cadmium levels in the lung, liver, kidney cortex, and urine samples from Australians without occupational exposure to metals. Arch. Environ. Health.

[13] D’Haese P.C., Couttenye M.M., Lamberts L.V., Elseviers M.M., Goodman W.G., Schrooten I., Cabrera W.E., De Broe M.E. (1999). Aluminum, iron, lead, cadmium, copper, zinc, chromium, magnesium, strontium, and calcium content in bone of end-stage renal failure patients. Clin. Chem..

[14] Carlsson L., Lundholm C.E. (1996). Characterisation of the effects of cadmium on the release of calcium and on the activity of some enzymes from neonatal mouse calvaria in culture. Comp. Biochem. Physiol. C Pharmacol. Toxicol. Endocrinol..

[15] Romare A., Lundholm C.E. (1999). Cadmium-induced calcium release and prostaglandin E2 production in neonatal mouse calvaria are dependent on cox-2 induction and protein kinase C activation. Arch. Toxicol..

[16] Suzuki Y., Morita I., Yamane Y., Murota S. (1989). Cadmium stimulates prostaglandin E2 production and bone resorption in cultured fetal mouse calvaria. Biochem. Biophys. Res. Commun..

[17] Marth E., Barth S., Jelovcan S. (2000). Influence of cadmium on the immune system. Description of stimulating reactions. Cent. Eur. J. Public Health.

[18] Marth E., Jelovcan S., Kleinhappl B., Gutschi A., Barth S. (2001). The effect of heavy metals on the immune system at low concentrations. Int. J. Occup. Med. Environ. Health.

[19] Hemdan N.Y., Emmrich F., Sack U., Wichmann G., Lehmann J., Adham K., Lehmann I. (2006). The in vitro immune modulation by cadmium depends on the way of cell activation. Toxicology.

[20] Burt B. (2005). Position paper: Epidemiology of periodontal diseases. J. Periodontol..

[21] Horiguchi H., Oguma E., Sasaki S., Miyamoto K., Ikeda Y., Machida M., Kayama F. (2005). Environmental exposure to cadmium at a level insufficient to induce renal tubular dysfunction does not affect bone density among female Japanese farmers. Environ. Res..

[22] Wood N., Johnson R.B. (2005). Recovery of periodontopathogenic bacteria from embalmed human cadavers. Clin. Anat..

[23] Karhunen V., Forss H., Goebeler S., Huhtala H., Ilveskoski E., Kajander O., Mikkelsson J., Penttila A., Perola M., Ranta H. (2006). Radiographic assessment of dental health in middle-aged men following sudden cardiac death. J. Dent. Res..

[24] Nishijo M., Nambunmee K., Suvagandha D., Swaddiwudhipong W., Ruangyuttikarn W., Nishino Y. (2017). Gender-Specific Impact of Cadmium Exposure on Bone Metabolism in Older People Living in a Cadmium-Polluted Area in Thailand. Int. J. Environ. Res. Public Health.

[25] Prozialeck W.C., Edwards J.R. (2012). Mechanisms of cadmium-induced proximal tubule injury: new insights with implications for biomonitoring and therapeutic interventions. J. Pharmacol. Exp. Ther..

[26] Apinan R., Satarug S., Ruengweerayut R., Mahavorasirikul W., Na-Bangchang K. (2010). The influence of iron stores on cadmium body burden in a Thai population. Environ. Geochem. Health.

[27] Vignery A., Baron R. (1980). Dynamic histomorphometry of alveolar bone remodeling in the adult rat. Anat. Rec..

[28] King G.J., Keeling S.D., Wronski T.J. (1991). Histomorphometric study of alveolar bone turnover in orthodontic tooth movement. Bone.

[29] Waddington R.J., Embery G., Last K.S. (1988). The glycosaminoglycan constituents of alveolar and basal bone of the rabbit. Connect. Tissue Res..

